# Four Decades After the Discovery of Regenerating Islet-Derived (Reg) Proteins: Current Understanding and Challenges

**DOI:** 10.3389/fcell.2019.00235

**Published:** 2019-10-22

**Authors:** Zijing Chen, Shawna Downing, Emmanuel S. Tzanakakis

**Affiliations:** ^1^Department of Chemical and Biological Engineering, Tufts University, Medford, MA, United States; ^2^Clinical and Translational Science Institute, Tufts Medical Center, Boston, MA, United States

**Keywords:** Reg proteins, pancreas, diabetes, pancreatic adenocarcinoma, gastrointestinal cancer

## Abstract

Regenerating islet-derived (Reg) proteins have emerged as multifunctional agents with pro-proliferative, anti-apoptotic, differentiation-inducing and bactericidal properties. Over the last 40 years since first discovered, Reg proteins have been implicated in a gamut of maladies including diabetes, various types of cancer of the digestive tract, and Alzheimer disease. Surprisingly though, a consensus is still absent on the regulation of their expression, and molecular underpinning of their function. Here, we provide a critical appraisal of recent findings in the field of Reg protein biology. Specifically, the structural characteristics are reviewed particularly in connection with established or purported functions of different members of the Reg family. Moreover, Reg expression patterns in different tissues both under normal and pathophysiological conditions are summarized. Putative receptors and cascades reported to relay Reg signaling inciting cellular responses are presented aiming at a better appreciation of the biological activities of the distinct Reg moieties. Challenges are also discussed that have hampered thus far the rapid progress in this field such as the use of non-standard nomenclature for Reg molecules among various research groups, the existence of multiple Reg members with significant degree of homology and possibly compensatory modes of action, and the need for common assays with robust readouts of Reg activity. Coordinated research is warranted going forward, given that several research groups have independently linked Reg proteins to diseased states and raised the possibility that these biomolecules can serve as therapeutic targets and biomarkers.

## Introduction

Since their discovery four decades ago, regenerating-islet derived (Reg) gene proteins have been linked to diverse pathologies including pancreatic ductal adenocarcinoma (PDAC), calcifying pancreatitis (De Caro et al., 1979), diabetes (Graf et al., 2006; Parikh et al., 2012) and extrapancreatic maladies such as Alzheimer disease and cancers of the gastrointestinal (GI) tract. Surprisingly though, very little is known about the regulation of their expression, exact functional role(s), and underlying mechanism(s). Even a receptor of rat Reg1 identified (Kobayashi et al., 2000) as homologous to the human exostosin-like glycosyltransferase 3 (EXTL3) (Kim et al., 2001) is still described in relevant literature as putative and its cellular localization and signaling role(s) are debatable (Osman et al., 2003). Sporadic studies have implicated various moieties such as the mitogen-activated protein kinase (MAPK) phosphatase (MKP-1) and phosphoinositide 3-kinase (PI3K) (Kadowaki et al., 2002; Takasawa et al., 2006; Mueller et al., 2008) as transducers of Reg signaling, but these results have been inconclusive or even conflicting.

Yet, Reg proteins are emerging as multifunctional moieties. Several Reg family members have been shown to promote proliferation, differentiation, and prevent apoptosis in diverse cell types in different contexts. For instance, PDAC and diabetes patients have higher serum levels of REG1A compared to healthy subjects, and REG3A is also increased in ductal fluid (Astorri et al., 2010; Porterfield et al., 2014; Yang et al., 2015; Li et al., 2016b). REG4 is overexpressed in colorectal and gastric carcinomas (Violette et al., 2003; Oue et al., 2005) whereas REG3A is expressed in hepatocellular carcinomas with β-catenin mutations (Cavard et al., 2006). Additionally, Reg proteins (e.g., human REG3A and mouse Reg3g) act as antimicrobial peptides contributing to the regulation of gut microbiota (Vaishnava et al., 2011; Mukherjee et al., 2014). Such associations make more pressing the need for better understanding the biology of Reg proteins and defining their functional features. Progress in this direction however, has been hampered by the existence of multiple members in the Reg family making challenging to gain insights into Reg regulation and bioactivity. This is further exacerbated by the inconsistent nomenclature for Reg proteins adopted in the published literature (Parikh et al., 2012).

In this review, we discuss the structural characteristics of Reg proteins as they may permit to shine light on Reg activity. The involvement of Reg members is also surveyed, particularly in various diseases. Signaling networks evidenced to operate in conjunction with Reg are also reviewed. Lastly, challenges are presented regarding research aiming to advance our knowledge of the expression and function of Reg proteins.

## Structure of Reg Proteins

**FIGURE 1:**
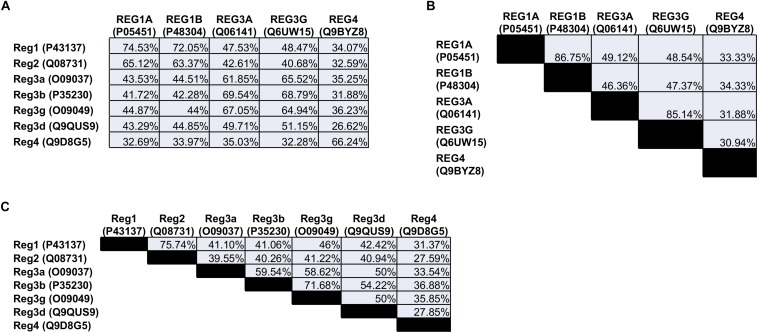
Amino acid sequence homology of the **(A)** human vs. the mouse Reg proteins, **(B)** human, and **(C)** mouse Reg proteins to each other. The numbers in parentheses refer to the individual protein entry ID in the UniProt database (http://www.uniprot.org). When multiple IDs are available for a protein, an ID is selected corresponding to an entry that has undergone review. Homology between sequences was quantified with the NCBI protein BLAST tool (https://blast.ncbi.nlm.nih.gov).

The Reg family comprises four groups (Reg1, Reg2, Reg3, and Reg4) of proteins based on their primary structure (Okamoto, 1999). Reg1 was discovered independently by various groups and reported under different names, such as lithostathine (due to the protein’s presumed inhibitory role in pancreatic stone formation), pancreatic stone protein (PSP; partially hydrolyzed form of Reg1), P19 and pancreatic thread protein (PTP) (Gross et al., 1985; Terazono et al., 1988; Watanabe et al., 1990). The Reg1 mRNA encodes a 166-amino acid (aa) preprotein including a 22-aa N-terminal signal sequence that is cleaved in the secreted protein (see below). Members of the first group are found in different species including humans and rodents. REG1A (also known as lithostathine) and REG1B are expressed in humans. The two proteins are 87% homologous to each other and exhibit a 72–74% similarity with the murine Reg1 (Figure 1).

The mouse Reg2 has the highest sequence similarity to Reg1 (76%) whereas there is no human ortholog of Reg2.

The Reg3 group includes the rodent Reg3a (or Reg3α), Reg3b (or Reg3β), Reg3g (or Reg3γ), and Reg3d (or Reg3δ). In contrast, only REG3A (also reported as hepatocarcinoma-intestine-pancreatic protein (HIP), pancreatitis-associated protein (PAP), and PAP1) and REG3G (also referred to as PAP1B) are expressed in human cells. REG3G is 65-66% homologous to the murine Reg3a, Reg3b and Reg3g. Analogously, REG3A is 62–67% similar to murine Reg3a and Reg3g, and 70% to Reg3b.

The last Reg group, Reg4, contains only one member. REG4, which was discovered more recently (Hartupee et al., 2001), is expressed in rodents and humans and exhibits the lowest similarity with any of the other Reg proteins. Overall, five Reg members are expressed in humans: REG1A, REG1B, REG3A, REG3G, and REG4.

Given the high homology among various Reg genes, it is very likely that all originated from the same ancestral gene (Hartupee et al., 2001). All Reg genes are composed of six exons except from the Reg4 gene which consists of seven exons. In fact, all human Reg genes are located on the second chromosome (2p12) whereas the gene for Reg4 is on chromosome 1 (1p11-3). In mice, the Reg1-3 groups are clustered on chromosome 6C3 and Reg4 is located on 3F3. The genes span two to three kilobases in length (Hartupee et al., 2001).

**FIGURE 2:**
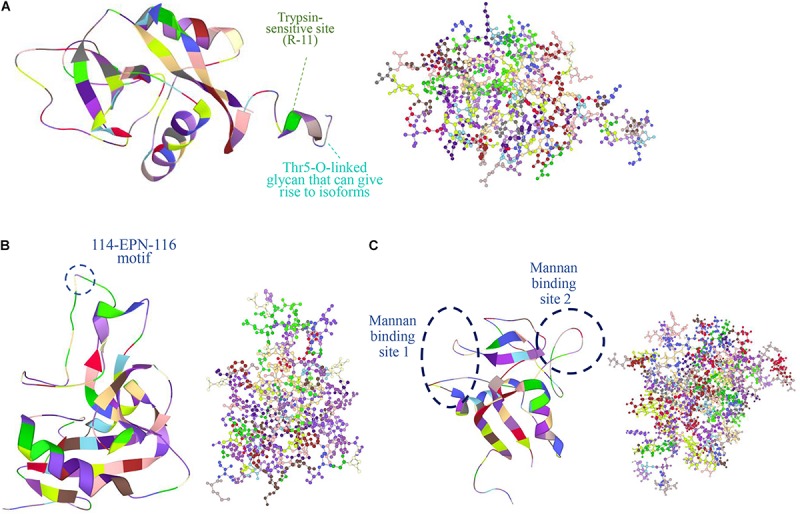
Protein structures obtained from the Protein Data Bank (PDB: https://www.rcsb.org/) are shown in figures of ribbon and ball and stick (colored by residues) of the human **(A)** REG1A (PDB ID: 1QDD, Gerbaud et al., 2000), **(B)** REG3A (PDB ID: 4MTH, Mukherjee et al., 2014), and **(C)** REG4 (PDB ID: 2KV3, Ho et al., 2010). In REG1A, the signal peptide is 22-aa long shortening the protein chain from 166 to 144 aa with a trypsin-sensitive arginine at position 11 (R-11) (De Reggi and Gharib, 2001). A Thr5-O-linked glycan is also featured that gives rise to different isoforms (De Reggi et al., 1995). REG3A (HIP/PAP) recognizes peptidoglycan carbohydrate backbones through an 114-EPN-116 motif that confers bactericidal activity while a E114Q mutation weakens this interaction (Lehotzky et al., 2010). REG4 has a C-type like domain (CTLD) and two calcium-independent sites that bind mannan (Ho et al., 2010). Images were created in LiteMol Suite (Sehnal et al., 2017).

Perhaps the most distinctive feature of Reg proteins is the C-type-like lectin domain (CTLD), which selectively binds a wide variety of ligands and carbohydrates (Dieckgraefe et al., 2002). Typically, Ca^2+^ mediates many CTLD interactions including binding to glycans. In other instances, Ca^2+^ causes a functional transformation of CTLD without binding carbohydrates. For example, the binding of Ca^2+^ to the CTLD of human tetranectin inhibits the protein’s interaction with plasminogen (Graversen et al., 1998). Based on structural analysis however, no Reg member contains a characteristic Ca^2+^-binding motif (Zelensky and Gready, 2005). Yet, Lee et al. (2003) reported the 1:1 binding of Ca^2+^ to recombinant human lithostathine produced in *E. coli* (thus, not being glycosylated; see below about Reg glycosylation). This interaction was attributed to the presence on the lithostathine surface of anionic residues (E30, D31, E33, D37, D72, and D73) and their mutation resulted in the significant decrease or loss of sugar binding affinity. Other Reg members are reported to bind carbohydrates in a Ca^2+^-independent manner. The human REG4 binds to mannan with two Ca^2+^-independent sites (Figure 2) (Ho et al., 2010). It also binds to heparin but not glucose, mannose and galactose (Ho et al., 2010). REG3A (HIP/PAP) also recognizes peptidoglycan through an 114-EPN-116 motif while a E114Q mutation weakens drastically this interaction (Lehotzky et al., 2010) (Figure 2). The same mutation reduces the bactericidal effect of REG3A against *L. monocytogenes* suggesting that REG3A selectively binds to bacterial cell surface sugars. Binding affinity increases with the length of the carbohydrate moiety, which is the principal factor (as opposed to the peptide sequence) dictating the REG3A/sugar interaction. This suggests that Reg combines selectively with peptidoglycans on the bacterial cell wall despite the presence of soluble peptidoglycan fragments. Like the REG3A, the murine Reg3g and Reg3b also feature the EPN motif and thus bind to peptidoglycan, chitin and mannan but not to dextran (Cash et al., 2006). In contrast, the murine Reg3a lacks this domain and does not bind peptidoglycan. It is plausible that Reg binding to sugars without calcium may serve the purpose of maintaining the carbohydrate recognition interaction in the low-pH milieu of the GI tract. Along similar lines, various Reg members (e.g., REG3A, Reg1, Reg3g) are reportedly involved in the agglutination of bacteria (Iovanna et al., 1993; Cash et al., 2006). Such function may serve to control microbiota during inflammation (Ogawa et al., 2003; Keilbaugh et al., 2005). Whether the presence of CTLD (i) imparts functional attributes to Reg proteins besides their bactericidal activity, and (ii) contributes to potential interactions with other molecules remain open questions.

The human lithostathine has a Thr5-O-linked glycan that gives rise to different isoforms (Figure 2). De Reggi et al. (1995) determined the sequence of seven lithostathine glycoforms out of 11 isolated. The glycoforms contain the same core structure GlcNAc(β1-6)[Gal(β1-3)]GalNAcα- with extensions of variable length. The importance of this diversity in glycosylated forms is unclear but the glycan chain is postulated to aid the stability of the protein and be involved in the localization of Reg protein.

A feature of Reg proteins is a cleavage site for trypsin near the N-terminus proceeded by a putative signal peptide. In human REG1A the signal peptide is 22 aa long shortening the protein chain from 166 aa to 144 aa with a trypsin-sensitive arginine at position 11 (Figure 2A) (De Reggi and Gharib, 2001). The cleavage site is displayed by most Reg protein members expressed in rodents and humans. Trypsin digestion results in a soluble short peptide and the remaining insoluble fragment of over 130 residues that forms fibrils resistant to further digestion by pancreatic proteases (except for rat PAP II/Reg3a which does not form insoluble precipitates and is digested by proteases after trypsin activation) (Graf et al., 2001). The latter resembles the action of pancreatic zymogens, which become active upon cleavage of a peptide sequence although the physiological role of formation of Reg fibrils remains unclear. The presence of these structures has been documented in pancreatic stones of patients with chronic pancreatitis. It was hypothesized that cleaved Reg protein forms plugs in the biliary tract after regurgitation of pancreatic juice with activated trypsin being present. In support of this hypothesis, cleaved Reg (PSP), trypsinogen and activated trypsin were detected in the bile-duct bile and the gallbladder bile of the patients but were absent in those of the controls (Ochiai et al., 2004). Interestingly, there was no evidence of the presence of Reg in the aforementioned plugs of patients with pancreaticobiliary maljunction, and the PSP concentration in the patients’ bile was three orders of magnitude lower than in the pancreatic juice under normal conditions. Human REG3A also assembles into filaments in the gut with a possible bacteriostatic role but the process requires the presence of cell membranes (Mukherjee et al., 2014).

It should be noted that lithostathine forms fibrils at neutral pH by undergoing autolytic cleavage via a yet poorly understood mechanism (Cerini et al., 1999). Parallels have been drawn between this process and the formation of Alzheimer plaques consisting of β-amyloid fibers as a result of the self-assembly of protein oligomers (De Reggi and Gharib, 2001). However, unlike typical amyloid fibrils, which have a cross-β-sheet structure, Reg fibrils display a secondary structure similar to that of the native protein (two α-helices and eight β-strands) (Ho et al., 2006). Nonetheless, PTP (lithostathine)-reactive brain lesions and elevated PTP expression are observed in Alzheimer patients (Ozturk et al., 1989; de la Monte et al., 1990). In fact, both lithostathine and PAP (Reg3) are present in senile plaques and neurofibrillary tangles of patients with Alzheimer disease (Duplan et al., 2001). The physiological role of Reg proteins in the disease remains elusive. Reg expression in the brain may be upregulated by inflammation similar to Reg mRNA induction with acute pancreatitis (Dusetti et al., 1996a). Increased Reg expression may lead to autolysis of the protein and formation of fibrils exacerbating the precipitation of β-amyloid peptides at the early stages of Alzheimer disease.

## Reg Protein Expression Patterns and Pathophysiology

### Pancreas

Reg proteins were initially discovered in the regenerating rat islets following pancreatectomy (Terazono et al., 1988), and while the majority of published reports pertain to their expression in pancreatic cells, Reg proteins have also been implicated in pathological conditions of the digestive tract (Hervieu et al., 2006) as well as of other tissues, for instance, of the nervous (Duplan et al., 2001) and reproductive systems (Du and Yao, 2013).

There is very little information to date about the Reg expression during development. The *REG1* mRNA exhibits a 20-fold increase in the human acinar pancreas after 16 weeks of gestation (Mally et al., 1994). The expression of PAP (REG3) genes is also detected in fetal pancreas, stomach, jejunum, and colon (Bartoli et al., 1998). During mouse development, the presence of Reg1 mRNA is detected on E9 just before pancreatic organogenesis (Perfetti et al., 1996b) and on E13 in the fetal intestine (Ose et al., 2007). To our knowledge, there is no information available on the expression of Reg genes at earlier times of embryonic development, although we have reported the expression of Reg proteins in murine embryonic stem cells in culture (Jing et al., 2010). It should be noted however that transgenic mice featuring knockouts of various Reg genes [e.g., Reg1 (Unno et al., 2002), Reg3g (Vaishnava et al., 2011); Table 1] are born at normal Mendelian ratios and they show normal growth indicating that Reg proteins are dispensable for development.

**TABLE 1:** Studies utilizing rodent models with Reg gene deletion of overexpression.

**Reg family type**	**Organ**	**Genotype**	**Treatment**	**Phenotype**	**References**
Murine Reg	Pancreas	*Reg* ^–/–^ (KO) mouse, insulin II promoter*-Reg* (TG) mouse	Goldthioglucose (GTG)-induced islet cell proliferation	Islet size is independent of Reg genotype. KO exhibits reduced thymidine incorporation; the average size of hyperplastic islets treated with GTG is decreased compared to control mice. β-cell thymidine incorporation is increased in TG; diabetes development is delayed in NOD mice.	Unno et al., 2002
Murine Reg1	Pancreas	*Reg1*^–/–^ mouse	Encephalomyocarditis (EMC) virus (D-variant) infection to induce β-cell damage and inflammation	*Reg1* was overexpressed in ATLANTIS and exocrine acinar cells around islets of wild-type (WT) mice after EMC infection. Thymidine incorporation in EMC virus-infected WT group is significantly higher than in EMC virus-infected KO mice and non-infected mice.	Aida et al., 2018
Murine Reg1	GI Tract	*Reg1^–/–^; Reg1^+/+^* mouse	N/A	KO mice show significantly reduced intensity of proliferation marker in the crypt and decreased migration cell speed to the villus tip.	Ose et al., 2007
Murine Reg2	Pancreas	*Reg2*^–/–^ mouse	Streptozotocin (STZ) to induce diabetes, caerulein to induce acute pancreatitis, high-fat diet	KO mice display no deficiency at young age (3–4 months) but have impaired insulin production and glucose tolerance later (13–14 months). No difference in the symptom severity in either STZ-induced diabetes (8- to 10-week-old), or caerulein-induced pancreatitis (6- to 8-week-old). After high-fat diet for 19 weeks, islet mass expansion and serum insulin level were reduced in KO vs. WT. Blood glucose is significantly lower in KO mice vs. WT from 11 to 19 weeks.	Li et al., 2017
Murine Reg2	Pancreas	Elastase-1 promoter-*Reg2* (TG) mouse for acinar cell-specific overexpression of *Reg2*	STZ-induced diabetes Caerulein-induced acute pancreatitis	TG shows no significant changes in Akt phosphorylation and levels of phosphoinositide 3-kinase, p85 and cyclin D1, normal islet growth and glucose homeostasis, and no protection against STZ-induced diabetes and caerulein-induced pancreatitis vs. WT.	Li et al., 2010
Murine Reg2	Liver	*Reg2*^–/–^ mouse; *Reg2*^+/+^ mouse	Fas intoxication with antibody J0−2; Partial hepatectomy	Sensitivity responding to Fas-induced oxidative stress is high in KO vs. WT. After partial hepatectomy, decreased survival rate and delayed liver mass regeneration is observed in KO vs. WT.	Lieu et al., 2006
Human REG3A	Liver	*REG3A*^+/+^ mouse (hepatocyte expression)	Dextran sodium sulfate (DSS) solution, or 2,4,6-trinitrobenzene sulfonic acid (TNBS) to induce colitis; Paraquat to induce oxidative stress	TG shows reduction in reactive oxygen species (ROS), increased survival of oxygen-sensitive commensal bacteria, changes in gut microbiota composition, resistance to DSS- and TNBS-induced Colitis. Fecal microbiota from TG mice protect non-TG mice from colitis.	Darnaud et al., 2018
Human REG3A	Liver	*REG3A*^+/+^ mouse (hepatocyte expression)	Partial hepatectomy; TNF-α and actinomycin D (ActD) to induce apoptosis	Liver regeneration is stimulated in TG as thymidine incorporation is significantly higher in TG vs. WT after partial hepatectomy. Primary TG hepatocytes show thymidine incorporation and higher viability vs. WT after treatment with TNF-α and ActD.	Simon et al., 2003
Reg3b Reg3g	GI Tract	*Reg3b*^–/–^ mouse *Reg3g*^–/–^ mouse; TG mouse with *Reg3g* expression only in intestinal epithelial and Paneth cells	Ethanol-induced liver disease	Compared to ethanol-fed WT, ethanol-fed *Reg3b* KO mice develop more severe liver injuries progressing from steatosis to steatohepatitis, and display an increased number of mucosa-associated bacteria.	Wang L. et al., 2016
				*Reg3g KO* mice show severe progression of ethanol-associated steatohepatitis, and display a higher number of mucosa-associated bacteria vs. WT but without a significant difference in the microbiota before or after ethanol feeding. Expression of the macrophage marker F4/80, hepatic expression of *Ccl2* and *Cxcl5*, and TNF-α are higher in ethanol-fed *Reg3b* and *Reg3g* KO mice. More Gram-negative bacteria in *Reg3b* KO than in WT. Microbiota, intestinal permeability and plasma levels of endotoxin are not significantly different in*Reg3b* KO. Ethanol-fed *Reg3g-*TG mice have significantly lower numbers of luminal bacteria, and less bacterial translocation vs. ethanol-fed WT.	
Reg3b	Pancreas	Rat insulin I promoter (RIP-I)-*Reg3*β mouse	STZ-induced diabetes	TG mice show partial protection against delayed onset of hyperglycemia and weight loss vs. WT after STZ treatment.	Xiong et al., 2011
Reg3g	GI Tract	*Reg3g ^–/–^* mouse	Bone marrow transplantation (BMT)	KO mice exhibit severe graft-versus-host disease (GVHD) and higher mortality after BMT compared to WT animals. Fecal microbiota composition does not differ between KO and WT either before BMT or during GVHD.	Zhao et al., 2018
Reg3g	GI Tract	*Reg3g*^–/–^ mouse	N/A	KO shows increased number of mucosal-associated Gram-positive bacteria, no difference in Gram-negative bacteria, and an increased activation of adaptive immunity vs. WT.	Vaishnava et al., 2011

*WT, Wild-type; KO, Knock-out; TG, Transgenic.*

Members of the Reg family are expressed primarily in normal pancreatic exocrine rather than endocrine cells (Michael et al., 1996; Baeza et al., 2001). Reg expression is age-dependent (Perfetti et al., 1996a; Martin et al., 2008; Wang et al., 2011) and increases during injury or inflammation. Indeed, various Reg proteins are linked to diabetes (Gurr et al., 2002; Martin et al., 2008; Astorri et al., 2010; Bacon et al., 2012; Cox et al., 2015; Yang et al., 2015), pancreatitis (Michael et al., 2000; Bluth et al., 2008; LaFonte et al., 2013) and pancreatic cancer (Grutzmann et al., 2003; Takehara et al., 2006; Legoffic et al., 2009; Park et al., 2011; Radon et al., 2015; Li et al., 2016b).

#### Diabetes

Increased circulating serum levels of REG1A are manifested across different types of diabetes including type 1 (T1D) (Astorri et al., 2010; Bacon et al., 2012), maturity onset diabetes of the young (MODY) (Astorri et al., 2010; Bacon et al., 2012) and type 2 (T2D) (Yang et al., 2015) indicative of β-cell apoptosis. T2D patients show upregulated Reg both at initial diagnosis and after the emergence of long-term complications. Reg levels in T2D and MODY, but not in T1D patients, correlate with the time since the onset of the disease (Wang et al., 2011; Bacon et al., 2012). T1D patients with increased REG1A exhibit significantly greater levels of corresponding autoantibodies than non-diabetic and T2D subjects (Astorri et al., 2010). After gastric bypass surgery, which allows to achieve glycemic control and reduce insulin resistance in T2D patients, upregulation of REG1A and REG3G may contribute to T2D remission (Sala et al., 2017).

A distinct type of acinar-like cell clusters touching Langerhans islets with thin interstitial surrounding (ATLANTIS) was reported in both healthy and diabetic pancreases (Aida et al., 2014) (Table 1). Those cell clusters were found to secret vesicles containing REG1A to neighboring islet cells in the non-diabetic human pancreas. In contrast, REG1A was overexpressed in ATLANTIS under diabetes (Aida et al., 2014). Furthermore, REG1 was overexpressed in ATLANTIS of *Reg1^–/–^* mice vs. their wildtype counterparts after encephalomyocarditis (EMC) virus infection characterized by β-cell damage and inflammation (Aida et al., 2018). This indicates that REG proteins are crucial in beta cell regeneration in this context.

In mice, knocking down *Reg* reduces the average islet size and thymidine incorporation (Unno et al., 2002). In contrast, transgenic non-obese diabetic (NOD) mice expressing *Reg* from the rat insulin II promoter (Ins-Reg) show increased β-cell regeneration and diminished hyperglycemia. Such findings and the reported mitogenic effects on β-cells (Michael et al., 1996; Borelli et al., 2005; Cox et al., 2015) support the notion that Reg proteins may serve as biomarkers (Astorri et al., 2010) and treatment targets for diabetes (Watanabe et al., 1994; Gross et al., 1998; Gurr et al., 2002; Li et al., 2010; Hou et al., 2011). For instance, administration of Reg protein induces β-cell mass expansion in female NOD mice (Gross et al., 1998) and rats with surgical diabetes (Watanabe et al., 1994). The overexpression of *Reg1* and *Reg3*β, but not *Reg2*, ameliorates streptozotocin (STZ)-induced diabetes in mice and accelerates recovery (Li et al., 2010; Hou et al., 2011; Xiong et al., 2011). *Reg2*^–/–^ mice display no deficiency at young age (3–4 months) but have impaired insulin production and glucose tolerance at a later age (13–14 months) (Li et al., 2017). Reg2 expression appears to contribute to obesity-induced islet compensation as *Reg2*^–/–^ mice show only 25% of the islet expansion exhibited by wild-type animals on high-fat diet (Table 1). The findings suggest that Reg2 expression is needed for islet expansion in aged mice or those on high-fat diet. It is worth noting that according to an earlier report (Wang et al., 2011) Reg2 peaks at 1 month, becomes undetectable after 3 months and is induced by obesity (Qiu et al., 2005) but its mechanism(s) of action remains unclear.

#### Pancreatitis

The expression of Reg1 and Reg3 in humans, mice and rats is linked to pancreatitis (Satomura et al., 1993; Michael et al., 2000; Bluth et al., 2008; LaFonte et al., 2013) as constituents of calciferous plugs manifested during the disease. In rodent models, Reg expression increases in chemical agent-induced pancreatitis or after pancreatitis surgery (Bluth et al., 2006, 2008). In addition, a significant elevation in Reg3 – but not Reg1 – is reported in the early phase of rat pancreatitis (Dieckgraefe et al., 2002). Pancreatic duct ligation lowers both the acinar cell mass and Reg1 production (Bluth et al., 2008). Other types of Regs have been probed in caerulein- or sodium taurocholate-induced pancreatitis. Reg2 overexpression appears to have little overall effect on pancreatitis (Li et al., 2010), whereas blocking of Reg1 and PAP-II with antibodies worsens the condition (Viterbo et al., 2009). These results are aligned with the general notion of Reg upregulation during inflammation.

#### Pancreatic Cancer

Reg proteins have also been investigated in the context of pancreatic cancer such as PDAC (Kleeff et al., 2016). Proliferating tumor cells in early stage PDAC express REG1A, REG1B, REG3A, and REG4 and throughout PDAC development (Takehara et al., 2006; Li et al., 2016b). The proteins are also elevated in the serum and urine of PDAC patients. Therefore, several groups suggested that Reg proteins may serve as biomarkers for early detection of pancreatic cancer (Grutzmann et al., 2003; Takehara et al., 2006; Legoffic et al., 2009; Park et al., 2011; Radon et al., 2015). Interestingly, higher levels of REG1A and REG1B are correlative with higher survival rates (Li et al., 2016b).

REG1A, REG3A, and REG3G expression is not observed in normal acini and ducts but is detectable in tumor-adjacent acinar areas (Zhou et al., 2010; Li et al., 2016b). Similarly, Reg3b is elevated in healthy acinar tissue near PDAC tumors and may interfere with the uptake of extracellular vesicles. Reg3b deficiency can also induce an alternative immune microenvironment in PDAC by reducing the M2/M1 ratio of tumor-associated macrophages and upregulating CD3^+^ cell infiltration through the activation of the STAT3 signaling pathway (see also section Signaling Pathways) (Gironella et al., 2013). However, the net effect of Reg3b on the pathology remains unclear. Limiting the uptake of vesicles may reduce the tumor volume near acinar cells and conversely increase tumor-produced extracellular vesicles throughout the bloodstream thereby enabling metastasis (Bonjoch et al., 2017). Reg3b and Reg3g reportedly promote the transition from pancreatitis to pancreatic cancer participation in the JAK2-STAT3 signaling axis (Loncle et al., 2015).

Introduction of recombinant REG4 enhances growth of a PDAC cell line in a dose-dependent manner. The use of a monoclonal antibody against REG4 counteracts the proliferation effect by inhibiting the REG4 autocrine/paracrine pathway and the subsequent phosphorylation of Akt (Takehara et al., 2006).

### Extra-Pancreatic Tissues

Reg proteins are implicated in cancer, inflammation or injury of tissues beyond the pancreas, including in the gastrointestinal (GI) tract, brain, liver and skin.

### GI Tract

In the GI tract, Reg proteins are expressed in the gastric mucosa, colon, and small intestine and have been linked to cell proliferation.

REG1A and REG1B are upregulated in human colonic or gastric mucosa with inflammatory bowel disease (IBD) (van Beelen Granlund et al., 2013) and ulcerative colitis (UC) (Tsuchida et al., 2017). Immunostaining of human colonic samples showed that REG1A correlated with TP53 (p53) overexpression in the development of UC-associated neoplasia (Tanaka et al., 2011) while a considerable number of p53-overexpressing high-grade dysplasias and invasive carcinomas were negative for REG1A. Interestingly, others have reported no significant relationship between p53 and REG1A in gastric cancers (Watanabe et al., 1990; Fukui et al., 2004; Sekikawa et al., 2005). Interleukins (ILs) in UC regulate Reg protein expression, particularly of REG1A that is stimulated by IL-22 and IL-6 (Sekikawa et al., 2010; Tsuchida et al., 2017), and of REG1B promoted by IL-22 (Tsuchida et al., 2017). It should be noted that IL-22 stimulation of REG1A is based on the presence of IL-activatable elements of the *REG1A* promoter and may be mediated through STAT3 tyrosine phosphorylation as in colon cancer cells *in vitro* (Sekikawa et al., 2010).

In celiac disease, serum levels of REG1A are over two-fold greater than normal but are reduced with the adoption of a gluten-free diet (Planas et al., 2011). Interestingly, REG1A appears to protect from bowel damage due to the use of non-steroidal anti-inflammatory drugs (NSAIDs), such as acetaminophen or ibuprofen. Increases in REG1A and REG1B are observed in acute amoebic colitis, a parasitic infection. In mice, Reg1 expression confers protection after exposure to *E. histolytica* (Peterson et al., 2011). Taken together, these findings support the surge in Reg proteins following GI tract damage caused by a multitude of factors.

Similar to other tissues, the link between Reg protein action and proliferation is suggested in the GI tract. Reg1 in the murine small intestine reportedly regulates growth that is essential for the generation and maintenance of the crypt-villus growth axis of the small intestinal mucosa, as *Reg^–/–^* mice shows significantly decelerated growth and migration of the intestinal epithelial cells to the villus tip (Ose et al., 2007). The pro-proliferative effect of Reg proteins may alleviate damages due to autoimmunity but could increase the risk of colon or gastric tumorigenesis. REG1A is associated with severe infiltration, poor prognosis and lymphatic invasion, but not with tumor size, tumor stage or p53 overexpression in gastric cancer as already mentioned (Watanabe et al., 1990; Fukui et al., 2004; Sekikawa et al., 2005). Increased REG1A is believed to promote cell growth or protect from apoptosis within tumors (Kadowaki et al., 2002; Sekikawa et al., 2005) but reports are conflicting. Knocking down REG1A (and REG3A) in gastric cancer cells leads to greater proliferation and infiltration whereas overexpression coincides with enhanced apoptosis (Qiu et al., 2017, 2018).

REG3A is expressed in the small intestine and is upregulated in IBD, possibly to attenuate inflammation and restore gut microbiota (van Beelen Granlund et al., 2013; Darnaud et al., 2018). REG3A appears to play a dual role in the intestine as a bactericidal agent and reducing apoptosis of epithelial cells. Upon digestion of the N-terminal pro-segment of REG3A by trypsin in the gut lumen, the protein binds to the surface peptidoglycans of Gram-positive bacteria, assembling into hexamers forming pores thereby compromising the membrane and eventually killing the bacterial targets (Mukherjee et al., 2014). The presence of the pro-segment potentially prevents unintended damage of the epithelial cells producing REG3A. Reg3b is expressed in murine intestinal epithelial cells and inhibits intestinal translocation of the Gram-negative *Salmonella enteritidi*s bacteria upon oral infection, but not of the Gram-positive *Listeria monocytogenes* (van Ampting et al., 2012). The expression of REG3G was predominantly observed in epithelial cells of the airway and intestine with a gradual increase during mouse development (Matsumoto et al., 2012). The antibacterial action of Reg3g mediates the spatial segregation of Gram-positive microbiota and the small intestinal epithelial surface (Vaishnava et al., 2011). *Reg3g^–/–^* mice display a higher number of mucosa-associated bacteria vs. their wild-type littermates but without a significant difference in the colon microbiota where Reg3g expression is low relative to that in the small intestine. However, overexpression of *Reg3g* in murine intestinal epithelial cells restricts bacterial colonization of mucosal surfaces and prevents bacterial translocation. In the same context, the presence of REG3G correlates with reduced steatosis and liver disease due to alcohol administration (Wang L. et al., 2016). Besides its antibacterial activity, REG3A serves as a survival signal to prevent apoptosis of crypt cells with graft-versus-host disease (GVHD) in allogeneic bone marrow transplantation (Zhao et al., 2018). Elevated serum REG3A is a strong indicator of GVHD in the GI tract with poor outcomes (Ferrara et al., 2011). *Reg3g^–/–^* mice exhibited severe GVHD and higher mortality after bone marrow transplantation compared to wild-type littermates. Administration of IL-22, which upregulates the expression of Reg3g (Zheng et al., 2008), prevented GHVD, increased Reg3g production in the ileum and decreased serum Reg3g levels but had no effect on *Reg3g^–/–^* mice. Incubation of TNF-α-treated HT-29 colorectal cells with recombinant REG3A reduced caspase cleavage and increased viability. Taken together, REG3A acts as a bactericidal and pro-survival signal in the intestinal milieu.

Another Reg member, Reg4, is expressed in UC tissues in correlative levels with basic FGF (bFGF) and hepatocyte growth factor (HGF) mRNA (Nanakin et al., 2007). REG4 was actually isolated from an inflammatory bowel disease cDNA library (Hartupee et al., 2001) and it is overexpressed in colon mucosa with Crohn’s disease (CD) (Nanakin et al., 2007; Tsuchida et al., 2017). Moreover, Reg4 has been identified as a marker of deep crypt secretory cells in Lgr^+^/CD24^+^ sorted cells, and Reg4^+^ cells promote organoid formation of single Lgr5^+^ colon stem cells (Sasaki et al., 2016). REG4 is thought to be an early stage serum biomarker of gastric cancer metastasis (Tao et al., 2011) and resistance to apoptosis after treatment with 5-fluorouracil (5-FU) used for chemotherapy (Mitani et al., 2007). REG4 expression is directly regulated by the CDX2 protein, an important transcriptional factor for development of intestinal epithelial cells, by binding to the 5′-flanking region of REG4 (Naito et al., 2012). In diffuse-type gastric carcinoma cells, TGF-β signaling inhibits REG4 expression in the cancer-initiating cells (Katsuno et al., 2012). Colorectal cancer patients with high tumor content of REG4 exhibit a poorer overall survival rate and disease-free survival rates compared to patients with tumors devoid of REG4 (Zhu et al., 2015). While the reason for this remains to be elucidated, REG4 appears to accelerate the transition through cell cycle promoting mitosis by upregulating the genes for cyclins D1 (*CCND1*) and D3 (*CCND3*), and for cyclin-dependent kinases *CDK4* and *CDK6* (Bishnupuri et al., 2014). In fact, REG4 knockdown induces apoptosis and inhibits the proliferation of colon cancer cells (Vanderlaag et al., 2012). This effect may be mediated by the transcription factor GATA6 regulating proliferation and development in the GI tract (Kawasaki et al., 2015). MicroRNA-363, which is reduced in colon tumors, suppresses *GATA6* leading to REG4 downregulation (Kawasaki et al., 2015).

#### Nervous System

Reg proteins are also involved in the pathophysiology of the nervous system. Reg1, Reg2/PAP1, PAPII, PAPIII and Reg4, are upregulated after injury of the motor neurons in rats (Namikawa et al., 2005) and PAPIII expression, specifically, is correlated with regeneration time (Namikawa et al., 2006). Reg1 is reported to stimulate neurite outgrowth through the EXTL-3 receptor in both mice and rats (Acquatella-Tran Van Ba et al., 2012). In animal models of tauopathy, Reg1 also regulates tau protein phosphorylation via the AKT/GSK3-β pathway only if tau is in a pre-phosphorylated state (Moussaed et al., 2018). PAP-1 and PAP-III proteins may protect against damaging factors in traumatic brain injury (TBI) and seizure, as PAP-1 is overexpressed in central neurons in human TBI patients, and PAP-1 and PAP-III are expressed in the hippocampal and parahippocampal areas after kainic acid-induced seizure in rat models (März-Weiss et al., 2011). HIP/PAP (REG3A) rescued neuronal death and glial activation after neuronal damaging factors *in vivo*, further suggesting that this Reg protein has neuroprotective/neuroregenerative effects (Haldipur et al., 2014). Rat PAP-III protein undergoes proteolytic N-terminal cleavage by trypsin-like protease(s) in injured sciatic nerves after axotomy, and mediates neurite elongation (Konishi et al., 2013). Besides brain injuries, Reg family proteins such as Reg1 and Reg3 are present in the plaques and neurofibrillary tangles of patients with Alzheimer disease. Greater levels of these Reg proteins are found in the cerebrospinal fluid of Alzheimer patients, compared to those of normal subjects and even of patients with multiple sclerosis (Duplan et al., 2001).

Rat Reg2 may prevent demyelization and promote regrowth of myelin after injury (Jiang et al., 2016). Reg2 is expressed in primary rat Schwann cells *in vitro* and *in vivo* in both sensory and motor neurons during development. This is stimulated by peripheral nerve axotomy in adult rats. Inhibition of Reg2 signaling activity by antisera hinders regeneration of Reg2^+^ axons (Livesey et al., 1997). Although the addition of recombinant Reg2 has a weak proliferation effect in Schwann cells *in vitro*, it is significantly mitogenic in a dose-dependent manner in the presence of forskolin, an activator of adenylyl cyclase (Livesey et al., 1997). During mouse development, transient expression of Reg3b is observed in specific subsets of motor neurons and primary sensory neurons during embryonic and perinatal stages (Matsumoto et al., 2012). *Reg3b* (the homolog of Reg2 in rats) deletion delays myelination in subsets of hypoglossal motor neurons in mice but does not induce motor neuron cell death (Tebar et al., 2008).

#### Liver

REG3A was initially identified as hepatocarcinoma-intestine-pancreas/pancreatic associated protein (HIP/PAP) due its elevated gene expression in human hepatocellular carcinoma (HCC) in comparison to normal liver tissues (Lasserre et al., 1994; Cervello et al., 2002). *Reg3a* is absent in normal adult hepatocytes, most likely due to a silencer region in its promoter that is inactive in hepatoma cells (Dusetti et al., 1996b). *Reg3a* is also not detected in human fetal liver or during development of post-implanted mouse embryos (Lasserre et al., 1999). The upregulation of REG3A correlates with early stage HCC and β-catenin mutation, and it is reported to be a downstream target of β-catenin mutation in the Wnt pathway *in vitro* (Yuan et al., 2005; Cavard et al., 2006). We also found that Reg1 expression is modulated by canonical Wnt signaling in murine embryonic stem cells (Jing et al., 2010). Taken together, these studies suggest that Reg expression is associated with hepatic cancer cell proliferation or differentiation during hepatocellular carcinogenesis.

Besides its role in HCC, REG3A acts an extracellular matrix (ECM)-targeted scavenger of reactive oxygen species (ROS) in a dose dependent manner (Moniaux et al., 2011) preventing ROS-induced-mitochondrial damage due to acetaminophen overdose (Lieu et al., 2005).

Another Reg member, REG1, is upregulated in newly formed bile ductular structures in a rat liver regeneration model (Wang et al., 2009). In *Reg2^–/–^* mice, liver regeneration is markedly delayed, sensitivity responding to Fas-induced oxidative stress is high, and the IL-6/STAT-3 pathway is active (Lieu et al., 2006).

Overall, while Reg protein activity increases during liver regeneration and disease, the physiological significance of this pattern warrants further investigation.

#### Other Cancer Types and Maladies

The presence of REG1A is reported in extrapancreatic cancer types including non-small cell lung cancer (Minamiya et al., 2008), breast cancer (Sasaki et al., 2008), bladder cancer (Geng et al., 2017) and colorectal sessile serrated adenoma (Okamoto et al., 2013). Despite the negative prognosis associated with REG1A (Minamiya et al., 2008; Sasaki et al., 2008; Hara et al., 2015; Geng et al., 2017), higher REG1A in thoracic esophageal squamous cell carcinoma is indicative of increased sensitivity to chemotherapy treatment (Sato et al., 2013) and lower levels of lymphatic permeation and vascular invasion (Aboshanif et al., 2016).

REG1A is also upregulated in the minor salivary glands of patients with Sjörgen’s Syndrome (SS), an autoimmune condition characterized by dryness of the eyes and mouth. The protein is believed to play a role in the regeneration of ductal cells in these tissues (Kimura et al., 2009).

Reg proteins are also implicated in wound healing. REG3A enhances keratinocyte proliferation and differentiation after skin injury by stimulation via IL-17 (Lai et al., 2012). Human REG3A or murine Reg3g are overexpressed in epidermal hyperproliferative keratinocytes in psoriasis (Lai et al., 2012).

Several Reg family mRNAs are also detected in the rat periosteum, which is a membrane tissue enveloping the outer surface of bones (Tohma et al., 2017). Reg1 gene expression is significantly augmented in the periosteum during the healing period after a significant bone fracture (Tohma et al., 2017). In periosteum-derived mesenchymal stem cells, IL-6 induces Reg expression and proliferation (Tohma et al., 2017). Interestingly, Reg expression is absent in the bone, bone marrow or muscles.

## Signaling Pathways and Reg Proteins

A widely accepted signaling mechanism for the action of Reg proteins is still lacking. Our review encompasses human Reg members and their rodent analogs while studies on Reg3d [INGAP – islet neogenesis-associated protein (Rafaeloff et al., 1997)] are not summarized due to space limitations.

### Putative Reg Receptors

The difficulty of establishing the molecular pathways associated with Reg stems in large part from the fact that receptors of Reg remain elusive. Using a rat islet cDNA expression library, Kobayashi et al. (2000) identified a putative receptor of rat Reg1. This receptor corresponded to the human EXTL3, a member of the exostosis family comprising glycosyltransferases contributing to heparan sulfate synthesis. Overexpression of EXTL3 increases the effect of REG1A on neurite outgrowth (Acquatella-Tran Van Ba et al., 2012) and the two proteins colocalize by immunostaining analysis of rat PC12 cells and primary hippocampal neurons. However, the Reg1/EXTL3 interaction has not been shown conclusively (e.g., by co-immunoprecipitation). In fact, findings from the same group that identified EXTL3 as a Reg receptor indicated that this is unlikely to act upstream of kinases believed to be targeted by Reg (Kadowaki et al., 2002). Moreover, in a yeast two-hybrid analysis focusing on binding partners of rat Reg1, none of the identified clones exhibited homology with the EXTL3 gene (Mueller et al., 2008) (see below). Importantly, there is no known signaling role for EXLT3. EXTL3 possibly induces NF-κB activity with TNF-α stimulation (Mizuno et al., 2001), and Reg1 expression is related to inflammatory responses but there is no established link between these observations.

Interactions of other Reg proteins with receptors are also debatable or unknown. The binding of REG3A to the epidermal growth factor receptor (EGFR) was suggested given that the two moieties colocalize upon immunostaining of SW1990 pancreatic adenocarcinoma cells and EGFR/REG3A complexes are detected by co-immunoprecipitation (Liu et al., 2015). Cells incubated with REG3A have lower expression of cyclin D1 when treated with the EGFR inhibitor Erlotinib. Additionally, blocking of EXTL3 did not affect the REG3A-induced cell proliferation suggesting that EXTL3 is not part of the REG3A cascade. The gp130 transmembrane protein of the JAK-STAT cascade is suggested as a Reg3β receptor (see Signaling Pathways) (Loncle et al., 2015). REG4 purportedly interacts with the G-coupled receptor 37 (GPR37) in gastric cancer cells to promote metastasis (Wang H. et al., 2016). These findings warrant extensive studies to conclusively demonstrate the reported interactions of Reg with the stated receptors and address whether these are cell-type specific or generally applicable.

### Signaling Pathways

Our knowledge of cascades relaying Reg signals remains incomplete and surveys of the relevant literature lead at times to conflicting conclusions. Induction of β-cell regeneration by Reg1 is reportedly mediated by the phosphoinositide-3-kinase (PI3K) acting on the transcriptional factor ATF2. This results in upregulation of cyclin D1, which is suppressed when RINm5F β-cells are treated with the PI3K inhibitors LY294002 and wortmannin. Moreover, murine *Reg*^–/–^ islets display decreased phosphorylated (active) ATF2 and retinoblastoma (Rb) proteins. Interestingly, inhibition of the MAPK kinases MEK1 and MEK2 does not change significantly the cyclin D1 promoter activity in β-cells. In another study (Kadowaki et al., 2002) however, Reg overexpression is shown to promote proliferation of gastric cancer cells via activation of the MEK1/2-targeted ERK1/2 but not p38 and JNK. Similarly, murine primary acinar cells treated with Reg3α exhibit higher levels of phosphorylated ERK1/2 (Li et al., 2016b). Whether these discrepancies about the role of ERK1/2 as mediator of Reg signals point to contextual differences is an open question. Reg1 binds to MKP-1 for regulation of cyclin D in rat ductal cells (Mueller et al., 2008). Here though, exposure to Reg1 increased the phosphorylation of not only ERK1/2 but also JNK and p38. Moreover, the reversal of the suppression of JNK phosphorylation by streptozotocin in MIN6 cells has been correlated with murine Reg2 (Liu et al., 2010).

The gp130-JAK2-STAT3 cascade is also reported to mediate the effects of Reg3b (Loncle et al., 2015). Systemic administration of recombinant Reg3b protein rescues PanIN formation in Reg3b-deficient mice and the same Reg is upregulated in cultured AR-42J (rat) acinar cells after treatment with IL17A. Neutralization with antibodies for Reg3b or IL17A inhibits PanIN development in Pdx1-Cre; Kras^*G*12*D*^; Reg3b^+/+^ mice. Reg3b signals through the gp130-JAK2-STAT3 axis promoting cell growth and resistance to apoptosis based on the decline in the number of Reg3b^+^ cells from the peritumoral region to PDAC lesions exhibiting poorly differentiated cells [although the pattern of Reg3b in this report was debated (Li et al., 2016a; Loncle et al., 2016)]. Along the same lines, REG3A was linked to the activation of the JAK2-STAT3 pathway in pancreatic cancer cell lines (Liu et al., 2015) and of the MAPK in acinar cells (Ferres-Maso et al., 2009). It is suggested that REG3A synergistically with interleukins (e.g., IL-6) promotes proliferation of pancreatic cancer cells via a REG3A-JAK2-STAT3 positive feedback loop. Further studies are warranted to shine light on the signaling mediated by REG3A and Reg3b signaling.

Murine *Reg3a* overexpression in MIN6 insulinoma cells leads to increased (1.8-fold) Akt phosphorylation and cyclin D1/cdk4 levels concomitant with enhanced proliferation rate (∼2-fold vs. cells transfected with empty vector) (Cui et al., 2009).

REG4 also phosphorylates AKT in addition to EGFR in gastric cancer cells (Kuniyasu et al., 2009) resulting in the increased expression of anti-apoptotic BCL-2, BCL-XL and BIRC5. The repertoire of phosphorylation targets for REG4 includes Erk, Hsp27 and GSK3β in colon and prostate cancer cell lines (Vanderlaag et al., 2012). In contrast to findings for other Reg members, there were no detectable changes in p38 MAPK and JNK upon REG4 stimulation or knockdown. Multiple receptor tyrosine kinases (RTKs) are impacted by REG4 although the nature of this effect depends on the cell type. Phosphorylation of the insulin and insulin-like growth factor receptors is stimulated in HCT116 cells but suppressed in PC3 and KM12 cells whereas EGFR phosphorylation is also decreased in PC3 cells but is unaffected in the other two lines. Knockdown of REG4 resulted in the induction of p21 and p27, reduction of S-phase cells and the lowering of the anti-apoptotic BCL-2. Given that other growth factors realize their effects through RTKs, a crosstalk or feedback is plausible between Reg4 and such ligands.

The phosphorylation of Akt and GSK-3β by REG4 is also shown for signaling through the β-catenin and TCF4 (Bishnupuri et al., 2014). Consequently, the Reg4-mediated activation of TCF4 results in the upregulation of cyclin D1 and D3 and their partners CDK4 and CDK6 enhancing the proliferation of human colorectal cancer cells. Phosphorylation of Akt by REG4 through the EGFR and activation of ERK1/2 and CREB reportedly trigger the polarization of macrophages to M2 phenotype (Ma et al., 2016). Taken together, these findings suggest that Reg4 acts on RTKs, such as the EGFR, causing the phosphorylation of Akt with downstream effects on genes controlling cell proliferation and apoptosis, particularly in GI cancer cells. The aforementioned studies give an incomplete picture of the role of Reg4 in normal cells but reveal candidate moieties for further investigation of Reg4 signaling.

## Challenges

An obvious hindrance in the field of Reg protein biology has been the use of multiple names to refer to specific Reg members or forms (whole vs. cleaved polypeptide sequence). This has imposed significant challenges on comparative analyses of findings from published studies thereby making arduous the advancement of knowledge about these proteins. Therefore, the use of standard Reg gene/protein nomenclature among different research groups is essential.

Additionally, the cross-reference and conceivably interchangeable use of human and rodent Reg homologs within the same work oftentimes exacerbates difficulties in interpreting results and contrasting them with those from other studies. For example, the human REG3A has been suggested as an ortholog of mouse Reg3b (e.g., ref., Loncle et al., 2016) based on amino acid sequence homology (69%; Figure 1). REG3A is also 67% homologous to murine Reg3g (Reg3gamma) and this similarity has been pointed out in other studies (e.g., refs. Vaishnava et al., 2011; Mukherjee et al., 2014). It should be noted that Reg3b and Reg3g share 70% of their amino acids but are not identical proteins. Whether Reg3b and Reg3g serve the same purpose possibly compensating each other in mice is presently unclear. More importantly, caution should be exercised when extrapolating findings for a particular Reg member from one species to a member(s) with partial sequence homology in a different organism.

The generation of wild-type and modified Reg proteins in a variety of hosts including *E. coli* (Lee et al., 2003; Cash et al., 2006), yeast (Li et al., 2003), mammalian (HEK293 or CHO) cells (Namikawa et al., 2006) and even transgenic mice (Christa et al., 2000) has aided significantly the research on various aspects of Reg protein biology. For instance, recombinant rat PAP2 protein produced in *E. coli* with either a GST or 6xHis tag (Viterbo et al., 2010) is detected by western blotting at the expected size, mediates bacterial aggregation and causes the upregulation of interleukins (IL-1α, -1β and -6) and TNF-α genes in rat NR8383 macrophages. It should be noted that various Reg members (e.g., lithostathine) feature glycosylation moieties, which may contribute to the protein function and therefore bacterial (lacking glycosylation) and yeast (typically mannose-rich glycosylation) systems may not be ideal for the production of recombinant Reg products with physiological activity. In most cases, the downstream purification rather than the expression of recombinant Reg in culture presents challenges, given the issues surrounding the solubility, aggregation and autocleavage of these proteins.

Reports involving the production or use of recombinant Reg proteins also make apparent the lack of standardized assays for testing the activity of these proteins. This stems mainly from our incomplete picture of the exact cellular responses triggered by Reg proteins and the underpinning mechanisms. The induction of IL genes in macrophages and bacterial aggregation mentioned have been viewed as processes resulting from Reg exposure as mentioned above. Given the role of Reg as pro-proliferative agent, the growth of cells in culture has been used as a readout of recombinant Reg bioactivity. However, these cells are typically transformed and thus highly proliferative making challenging the accurate assessment of proliferation changes elicited by a particular Reg. Moreover, the presence of serum in the maintenance medium may confound any effects solely due to Reg. The anti-apoptotic role of Reg is also exploited in activity assays as a reduction in the loss of cell viability and caspase 3/7 activity in response to a cytotoxic agent (Loncle et al., 2015). The phosphorylation of ATF2 and activation of cyclin D1 promoter activity as downstream effectors of Reg have also been probed in a limited number of studies (Takasawa et al., 2006). The quantification of outgrowth of primary neurons or respective lines (e.g., N2a, Acquatella-Tran Van Ba et al., 2012) in culture after exposure to recombinant Reg has also been employed in checking the activity of laboratory-produced and commercially available recombinant Reg proteins. The mechanism of Reg-induced neurite extension is still unsettled. The assay outcome is very sensitive to the culture conditions including cell seeding density, type of matrix used for cell attachment, and regimen for changing media. To this end, the development of standardized assays with robust readouts and amenable to validation for determining Reg bioactivity will be essential for further progress in this field. Such assays will reduce the variability in the activity of different batches of recombinant Reg proteins prepared commercially or by individual laboratories.

Lastly, the potential regulation of Reg gene expression by microRNAs (miRNAs) has been largely unexplored. Recently, the murine *reg1*, which displays a 3′-UTR region that matches the seed of miRNA-7 (miR-7), was shown to be a direct target of this miRNA (Downing et al., 2019). Interestingly enough, miR-7 expression is higher in the islets than in exocrine cells (200:1) (Bravo-Egana et al., 2008; Correa-Medina et al., 2009) whereas Reg1 is abundant in acinar cells but not in normal islets. Because miR-7 has roles in β-cell proliferation and function, avenues are opened for exploring possible connections with the regulation of *reg1* expression and potentially shining more light on Reg function. The same study (Downing et al., 2019), however, demonstrated that unlike *reg1*, the human *REG1A* and *REG1B* are not targeted directly by miR-7. This exemplifies further that regulation of Reg expression may not be universal but species-dependent. To this date, there have been no reports of miRNA regulation of the expression of human Reg genes.

In summary, Reg proteins appear to assume multiple roles as pro-proliferative, anti-apoptotic and bactericidal agents. This repertoire warrants further research to expand our knowledge on the regulation and functional attributes of each Reg member, and potential synergies among distinct Reg proteins. Expediting research progress in this area will require addressing challenges including the ones discussed above. Given that several reports have linked Reg moieties to a gamut of maladies, particularly of the pancreas and GI tract, the idea is appealing that these proteins may serve as therapeutic targets and biomarkers.

## Author Contributions

ZC, SD, and ET researched the relevant literature, wrote the manuscript and approved its submitted form.

## Conflict of Interest

The authors declare that the research was conducted in the absence of any commercial or financial relationships that could be construed as a potential conflict of interest.
